# GPR183-mediated immune cell positioning and function: an integrative perspective across ligand gradients, tissue environments, and disease stages

**DOI:** 10.3389/fimmu.2026.1816598

**Published:** 2026-10-06

**Authors:** Xinrui Lin, Xinyu Dong, Zhe Jiang, Xuekun Xing

**Affiliations:** 1 School of Public Health, Guilin Medical University, Guilin, China; 2 Guangxi Key Laboratory of Environmental Exposomics and Entire Lifecycle Health, School of Public Health, Guilin Medical University, Guilin, China

**Keywords:** 7α,25-dihydroxycholesterol, disease-stage dependence, GPR183, immune-cell migration, ligand gradient, tissue microenvironment

## Abstract

G protein-coupled receptor 183 (GPR183), also known as EBI2, is a chemoattractant receptor whose principal endogenous ligand is 7α,25-dihydroxycholesterol (7α,25-OHC). Its expression varies dynamically across immune-cell subsets and activation states. By sensing local 7α,25-OHC gradients, GPR183 converts tissue metabolic cues into spatial information, thereby regulating immune-cell migration, tissue distribution, and function. Dynamic receptor expression, ligand synthesis and degradation, responding cell type, and the tissue microenvironment collectively shape the direction of pathway activity, whereas intervention studies in animals and longitudinal observations establish disease stage as a temporal coordinate for interpreting divergent effects. Genetic, pharmacological, and cell-engineering studies provide functional evidence that GPR183 contributes to lymphoid-tissue compartmentalization, host defense, inflammatory responses, tissue repair, and tumor-immune remodeling. Analyses of patient tissues and clinical cohorts extend this mechanistic framework to associations with disease activity, treatment response, and prognosis. Three intervention strategies have emerged: receptor agonism, pharmacological inhibition, and immune-cell engineering. The oral antagonist IPG11406 has entered early clinical evaluation, whereas preclinical assessment of other candidate strategies has focused on ligand stability, tissue selectivity, and long-term immune safety. This review integrates current evidence across four dimensions - immune-cell type, the 7α,25-OHC gradient, tissue microenvironment, and disease stage - and organizes the disease-related actions of GPR183 and potential stratified interventions around pathogenic recruitment, protective recruitment, tissue repair, tumor-immune remodeling, and biomarker applications.

## 1 Introduction

GPR183 was first identified in Epstein-Barr virus-infected B cells and was subsequently classified as a member of the G protein-coupled receptor family (1). In 2011, two independent studies identified 7α,25-dihydroxycholesterol (7α,25-OHC) as a potent endogenous ligand of GPR183 and demonstrated that it induces the migration of immune cells expressing this receptor (2, 3). 7α,25-OHC is generated sequentially by CH25H and CYP7B1 and is inactivated by HSD3B7. The differential distribution of these enzymes across stromal-cell compartments can generate spatially directed ligand signals (4).

The position of immune cells within tissues directly affects antigen recognition, intercellular contact, and subsequent effector responses. GPR183 does not provide a constitutive migratory cue of fixed direction; its effects also depend on dynamic changes in receptor expression. Mature B cells upregulate GPR183 early after activation and migrate toward the outer follicle and interfollicular regions, after which receptor downregulation enables a subset of cells to access the follicular center and participate in the germinal-center response (5–7). At the signaling level, GPR183 couples primarily to Gi/o proteins, whereas β-arrestin coupling contributes to cell migration, which is not strictly dependent on receptor internalization (8). Receptor abundance, ligand gradients, and signal adaptation therefore act together to determine GPR183-mediated positioning.

As investigation has expanded from lymphoid organs to inflamed tissues, injury settings, and tumor microenvironments, the GPR183 literature has come to encompass several levels of evidence. Genetic deletion, cell-transfer experiments, and pharmacological interventions primarily define functional roles, whereas expression profiling in patient tissues, transcriptomic analyses, and prognostic models establish relevance to human disease. Reorganizing this evidence by responding cell, tissue environment, and disease stage provides a clearer explanation for divergent outcomes of the same pathway than a disease-by-disease catalogue.

Accordingly, this review examines the direction of GPR183 signaling across four dimensions: responding cell, ligand gradient, tissue microenvironment, and disease stage. It distinguishes mechanistic evidence, engineering-based validation, and clinical associations, and uses this framework to discuss stratified interventions and key measures for translational evaluation (**Figure 1**).

**Figure 1:**
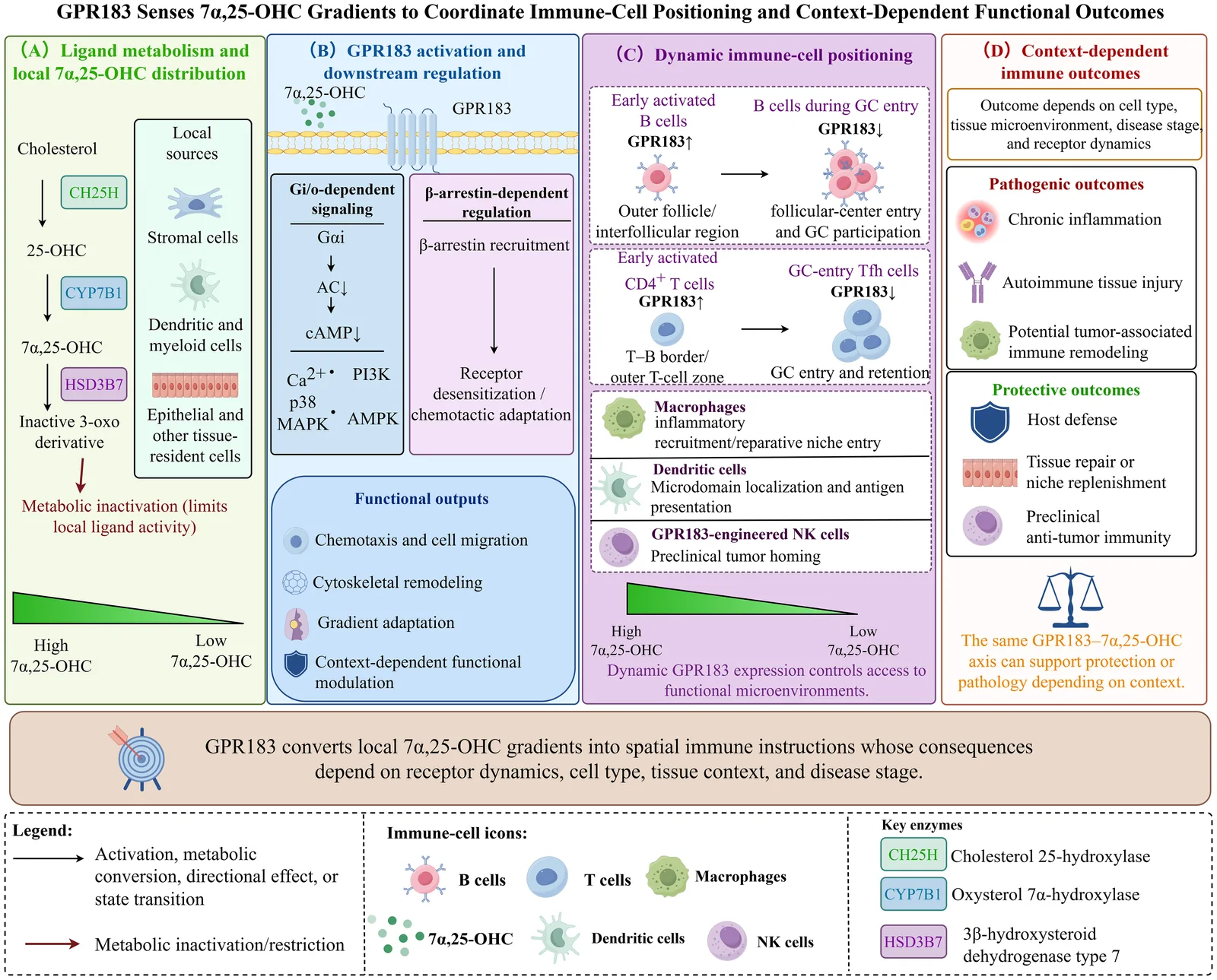
Graphical overview of GPR183 sensing of 7α,25-OHC gradients and the coordination of immune-cell positioning and context-dependent functions (created with Figdraw). **(A)** Local ligand metabolism and gradient formation. Cholesterol is converted by CH25H to 25-OHC and subsequently by CYP7B1 to 7α,25-OHC. HSD3B7 inactivates the ligand and thereby restricts its local range of action. Stromal cells, dendritic cells, myeloid cells, epithelial cells, and other tissue-resident cells serve as ligand sources in different tissues. **(B)** GPR183 activation and downstream regulation. GPR183 senses local 7α,25-OHC and initiates Gi/o-dependent signaling and β-arrestin-associated regulation, thereby influencing cell migration, cytoskeletal remodeling, gradient adaptation, and other context-dependent functions. **(C)** Dynamic immune-cell positioning. Early after activation, B cells and CD4^+^ T cells upregulate GPR183 and localize to the outer follicle/interfollicular region and the T-B border, respectively. Subsequent downregulation of GPR183 allows subsets of B cells and T follicular helper (Tfh) cells to enter and remain within germinal centers. Macrophages, dendritic cells, and engineered natural killer (NK) cells further illustrate distinct roles for this pathway in inflammatory recruitment, niche repopulation, antigen presentation, and preclinical tumor homing. **(D)** Context-dependent immune outcomes. The same GPR183-7α,25-OHC axis may contribute to host defense, tissue repair, niche repopulation, and preclinical antitumor immunity, but may also participate in chronic inflammation, autoimmune tissue injury, and potential tumor-associated immune remodeling. The direction of its effects is determined by the responding cell, tissue microenvironment, disease stage, and receptor dynamics. The green triangle narrows from high to low 7α,25-OHC activity. Black arrows denote activation, metabolic conversion, directional effects, or state transitions, whereas red arrows indicate ligand inactivation or restriction of an effect. 25-OHC, 25-hydroxycholesterol; 7α,25-OHC, 7α,25-dihydroxycholesterol; AC, adenylyl cyclase; AMPK, AMP-activated protein kinase; cAMP, cyclic adenosine monophosphate; CH25H, cholesterol 25-hydroxylase; CYP7B1, cytochrome P450 family 7 subfamily B member 1; GC, germinal center; Gi/o, inhibitory G-protein family; GPR183, G protein-coupled receptor 183; HSD3B7, hydroxy-Δ5-steroid dehydrogenase, 3β- and steroid Δ-isomerase 7; NK, natural killer; PI3K, phosphoinositide 3-kinase; Tfh, T follicular helper. The figure was created with Figdraw.

## 2 Literature search strategy

This narrative review was based primarily on literature retrieved from PubMed and Web of Science. The databases were searched from inception through August 2026 using combinations of the following terms: “GPR183”, “EBI2”, “7α,25-dihydroxycholesterol”, “7α,25-OHC”, “immune cell migration”, “immune positioning”, “tumor microenvironment”, “inflammation”, and “tissue repair”. ClinicalTrials.gov was also searched using “GPR183”, “EBI2”, and “IPG11406” to verify the clinical development status of GPR183-directed interventions; trial status was last checked on 16 August 2026. Studies were included primarily on the basis of their relevance to GPR183 biology, signaling mechanisms, disease associations, and therapeutic potential.

## 3 Heterogeneity of GPR183 expression and function across immune cells

### 3.1 Effects on B-cell migration, positioning, and function

The influence of GPR183 on B-cell positioning changes over the course of an immune response. In murine secondary lymphoid organs, 7α,25-OHC activity is relatively high in the outer follicle and interfollicular regions and relatively low in the follicular center (4). Following antigen stimulation, B cells initially upregulate GPR183 and redistribute toward the outer follicle, interfollicular regions, and splenic bridging channels, thereby supporting extrafollicular plasmablast differentiation and rapid antibody responses (6, 7).

In contrast to this early function, timely GPR183 downregulation is required for activated B cells to enter the follicular center and participate in the germinal-center response. In murine genetic models, GPR183-deficient activated B cells fail to access the outer follicle efficiently and instead show a follicular-center-biased distribution, whereas sustained or ectopic expression retains cells in the outer follicle and impairs their participation in germinal centers (6, 7). These interventions reveal two directionally distinct positioning defects: loss of GPR183 primarily disrupts early outer-follicular localization and plasmablast responses, whereas failure to downregulate the receptor prevents the transition from peripheral follicular regions to the follicular center. A more accurate model is therefore that early GPR183 upregulation supports the outer-follicular positioning of activated B cells and rapid extrafollicular antibody production, whereas later receptor downregulation releases a subset of activated B cells from outwardly directed 7α,25-OHC chemotactic cues, enabling entry into the follicular center and participation in germinal-center responses (6, 7). This temporal transition is also transcriptionally regulated. Through its RD2 domain, BCL6 recruits HDAC2 and represses *Gpr183* and *S1pr1* in germinal-center B cells. Disruption of BCL6 RD2 function dysregulates both migratory receptors and is accompanied by abnormal migration of activated B cells and impaired early germinal-center clustering (9).

The principle that local ligand sources regulate humoral immunity extends to terminally differentiated plasma cells. In the small-intestinal lamina propria, dietary cholesterol absorption and commensal recognition promote 7α,25-OHC production by duodenal epithelial cells. Gut plasma cells sense this local metabolic cue through GPR183, coupling their tissue position to the regulation of IgA secretion. In murine models, including epithelial-cell-specific *Ch25h* deletion, attenuation of epithelial 7α,25-OHC production releases the restraint on IgA secretion against commensal- and pathogen-derived antigens (10). Local ligands of nonhematopoietic origin thus connect plasma-cell positioning with mucosal antibody output.

In diseased mucosal tissues, GPR183/EBI2 expression is also associated with the composition and maturation state of antibody-secreting cells. Compared with tonsils, human nasal polyps analyzed in 2024 contained an EBI2^+^ B-cell population with higher proportions of total IgG- and anti-double-stranded-DNA IgG-secreting cells, while antibody-secreting cells as a whole exhibited a more mature plasma-cell transcriptional profile (11). This tissue-level comparison links EBI2^+^ cells to extrafollicular antibody responses in nasal polyps; direct perturbation will be required to define the functional contribution of the receptor itself.

The regulation of B-cell migration and function by GPR183 is not invariant in autoimmune settings. The proportion of GPR183^+^ B cells is reduced in patients with systemic lupus erythematosus (SLE) and in mice with established disease, without an evident sex difference. By contrast, during the initiation of lupus-like disease, increased splenic GPR183^+^ B-cell abundance and enhanced migration toward 7α,25-OHC occur only in female mice. Consistent with this pattern, early GPR183 blockade reduces B-cell activation and ameliorates disease only in female mice; comparable effects are not observed in male mice or at later disease stages (12). This temporal comparison places the GPR183-dependent B-cell response within early disease and a female biological context: human data define a disease-associated expression pattern, whereas intervention in mice provides corresponding functional evidence. Changes in GPR183 expression should therefore be interpreted together with cellular differentiation state, local ligand source, disease stage, and biological sex.

In extrafollicular B-cell responses, GPR183 may also participate in a spatial program distinct from entry into the germinal center. Age-associated B cells (ABCs) from patients with SLE display higher surface EBI2 expression and stronger ligand-directed migration. In lupus-prone mice, EBI2 contributes to the positioning of ABCs within extrafollicular regions of splenic bridging channels and is associated with the formation of pre-plasmablast ABCs and the production of autoreactive plasmablasts; B-cell-specific *Ebi2* deletion attenuates this process (13). The influence of GPR183 on B-cell fate can therefore be understood in terms of spatial differentiation routes: timely receptor downregulation favors entry of activated B cells into the follicular center, whereas specific extrafollicular responses use GPR183-mediated positioning to initiate a distinct effector program.

### 3.2 Regulation of the spatial positioning and function of T-cell subsets

The most informative evidence identifies spatial positioning as a central function of GPR183 in T cells, with effects on differentiation and effector responses frequently linked to redistribution across tissue microdomains. In murine lymph nodes, GPR183 biases naive CD4^+^ T cells toward peripheral regions of the paracortex, aligning them spatially with conventional type 2 dendritic cells (cDC2s), which are efficient at MHC class II antigen presentation. Loss of GPR183 abolishes this regional distribution of CD4^+^ T cells and delays antigen recognition, clonal expansion, differentiation, and the provision of help to other immune cells (14).

The effect of GPR183 on T follicular helper (Tfh) cell positioning changes with the stage of the immune response. Shortly after antigen activation, CD4^+^ T cells upregulate GPR183 and are guided by 7α,25-OHC signals to the B-cell follicle-T-cell zone interface and the outer T-cell zone. In this microenvironment, activated CD4^+^ T cells encounter activated dendritic cells expressing high levels of ICOS ligand (ICOSL) and CD25. These dendritic cells provide ICOSL-dependent costimulation that promotes Tfh differentiation and use membrane-bound and soluble CD25 to bind local IL-2, thereby reducing the inhibitory effect of IL-2 on Tfh differentiation. Accordingly, T-cell-specific GPR183 deficiency reduces Tfh-cell, plasma-cell, and germinal-center responses in mice (15). Once germinal centers form, Tfh cells must downregulate GPR183 to enter and remain within the germinal center, whereas cells retaining relatively high GPR183 expression are found more frequently in the follicular mantle (16). In human tonsillar germinal-center Tfh cells, BCL6 ChIP-seq identified binding at the GPR183 locus, and EBI2 expression was reduced (17), consistent with receptor downregulation during the germinal-center phase.

GPR183 can regulate T-cell function by controlling the intratissue positioning of already differentiated cells without necessarily altering subset differentiation. In a 2026 murine model of pulmonary *Cryptococcus neoformans* infection, an expanded population of CH25H^+^Ly6C^-^ macrophages produced 25-hydroxycholesterol, which was further converted within the tissue to 7α,25-OHC and directed GPR183-expressing Th2 cells toward infectious foci. Th2-cell-specific GPR183 deletion did not substantially alter pulmonary Th2-cell numbers, differentiation phenotype, or IL-13 expression, but disrupted localization within lesions and restored the responsiveness of Ly6C^+^ inflammatory macrophages to IFN-γ, as reflected by increased iNOS expression and reduced pulmonary fungal burden (18).

Human studies currently provide more evidence of treatment-associated expression changes than of tissue positioning. In a 2025 longitudinal cohort of patients with severe asthma, mepolizumab treatment reduced GPR183 expression in circulating ILC2, Th2, and Tc2 cells, whereas these cells showed greater capacity to produce IL-5 and IL-13 after *ex vivo* stimulation (19). This cohort documented parallel post-treatment changes in GPR183 expression and *ex vivo* effector responses; it defined a treatment-associated cellular state rather than migration or tissue localization.

### 3.3 Regulation of dendritic-cell migration and antigen presentation

The regional distribution of dendritic cells (DCs) after entry into secondary lymphoid organs affects their access to antigens and naive T cells and contributes to the initiation of adaptive immune responses. In the spleen and other lymphoid tissues, the spatial distribution of conventional dendritic cells is controlled by multiple chemotactic and retention cues. GPR183 contributes to the homeostasis and positioning of splenic cDC2s and acts together with S1PR1, CCR7, and integrins to organize these cells within the splenic microenvironment (20, 21).

Following antigen encounter, local 7α,25-OHC signals can further regulate GPR183-dependent redistribution of dendritic cells between tissue microdomains (20, 21). Migratory programs differ among dendritic-cell subsets and maturation states. Direct evidence currently supports GPR183-mediated positioning of splenic cDC2s, whereas migration of DCs from peripheral tissues to draining lymph nodes remains governed predominantly by CCR7-related signals (21, 22).

Dendritic-cell migration is also shaped by the local inflammatory milieu. Under homeostatic conditions, CCR7 is the principal mediator of DC migration from peripheral tissues to draining lymph nodes. In type 2 inflammatory settings, including parasitic infection and allergic inflammation, DCs may retain some migratory capacity even when CCR7 expression is reduced (22, 23). Lipid mediators such as eicosanoids, sphingolipids, and 7α,25-OHC may provide supplementary migratory cues when canonical chemotactic signals are altered (23). GPR183 and CCR7 therefore operate at different levels of dendritic-cell migration: CCR7 predominantly directs transit from peripheral tissues to draining lymph nodes, whereas GPR183 refines intratissue positioning in specific DC subsets and inflammatory contexts.

### 3.4 Functions in monocytes/macrophages and other innate and innate-like lymphocytes

Beyond B cells, T cells, and dendritic cells, GPR183 regulates monocytes, macrophages, and several innate and innate-like lymphocyte populations in barrier tissues. Across these cell types, however, GPR183 may contribute to migration, tissue positioning, or differentiation, and both the specific functional output and the strength of evidence vary with cell type and experimental model.

In monocytes and macrophages, GPR183 contributes to both cell-intrinsic antimicrobial responses and tissue recruitment. Activation of GPR183 by 7α,25-OHC enhances autophagy in monocytes and restricts the intracellular growth of *Mycobacterium tuberculosis* (24). In a murine model of M. tuberculosis infection, pulmonary CH25H and CYP7B1 expression increased together with 25-OHC abundance and GPR183 expression. Early macrophage recruitment was correspondingly reduced in GPR183-deficient mice, linking the GPR183/oxysterol axis to macrophage entry into infected lung tissue (25). In influenza and SARS-CoV-2 infection, genetic deletion or pharmacological blockade of GPR183 likewise reduces pulmonary macrophage infiltration (26, 27).

In intestinal innate lymphoid cells, GPR183 primarily regulates the spatial positioning of group 3 innate lymphoid cells (ILC3s). ILC3s with a lymphoid-tissue-inducer phenotype express high levels of GPR183 and migrate toward 7α,25-OHC (28, 29). This positioning promotes ILC3 accumulation in gut-associated lymphoid tissues and contributes to the formation of colonic cryptopatches and isolated lymphoid follicles (28–30).

Among innate-like lymphocytes associated with barrier tissues, GPR183 also contributes to the development and maintenance of IL-17-producing γδ T cells. Developing γδ thymocytes sense oxysterol signals produced by thymic medullary epithelial cells through GPR183, promoting Tγδ17-cell maturation. After these cells enter the skin, CH25H-dependent signals from basal keratinocytes continue to support this population (31).

## 4 Formation and sensing of the 7α,25-OHC ligand gradient

### 4.1 Metabolic production of 7α,25-OHC and gradient formation

Among the endogenous GPR183 ligands identified to date, 7α,25-OHC is the most potent. Two independent studies used ligand screening, receptor-signaling assays, and chemotaxis experiments to show that 7α,25-OHC activates GPR183 with high affinity and induces directional migration of receptor-expressing immune cells (2, 3). In the canonical biosynthetic pathway, cholesterol is first converted by cholesterol 25-hydroxylase (CH25H) to 25-hydroxycholesterol (25-OHC), which is subsequently converted to 7α,25-OHC by cytochrome P450 family 7 subfamily B member 1 (CYP7B1). Hydroxy-Δ5-steroid dehydrogenase, 3β- and steroid Δ-isomerase 7 (HSD3B7) converts 7α,25-OHC into a 3-oxo derivative that lacks agonist activity at GPR183, thereby terminating ligand signaling (2–4).

In secondary lymphoid organs, differential expression of ligand-generating and ligand-inactivating enzymes across stromal-cell subsets contributes to the establishment of 7α,25-OHC gradients. CH25H and CYP7B1 are relatively abundant in peripheral follicular regions, whereas CH25H expression is suppressed in follicular dendritic cells at the follicular center; HSD3B7-mediated ligand inactivation further restricts local activity. GPR183 ligand activity is therefore higher in the outer follicle and interfollicular regions than in the follicular center (4). Consistent with this distribution, *Ch25h* deletion or pharmacological inhibition of CYP7B1 reduces splenic GPR183 ligand activity and disrupts the normal positioning of activated B cells (2–4).

Metabolic-enzyme expression, tissue ligand abundance, and the spatial gradient represent three distinct levels at which pathological changes in GPR183 ligand biology can be evaluated. CH25H or CYP7B1 expression defines the potential for ligand generation, 7α,25-OHC measured in tissue homogenates reflects total ligand burden, and the combined assessment of ligand activity, responding-cell position, and pathway perturbation is required to infer changes in spatial gradients and their functional consequences.

### 4.2 Ligand sensing, Gi signaling, and adaptive regulation of GPR183

GPR183 detects local differences in 7α,25-OHC concentration and converts them into chemotactic signals for immune cells (2, 3). Binding of 7α,25-OHC stabilizes the active receptor conformation and promotes coupling to Gi proteins. Cryo-electron microscopy of the 7α,25-OHC-GPR183-Gi complex has further defined the structural basis of ligand binding, receptor activation, and G-protein recognition (32). In the canonical Gi pathway, Gαi inhibits adenylyl cyclase and lowers intracellular cyclic adenosine monophosphate (cAMP); early pharmacological studies likewise showed that GPR183 suppresses forskolin-induced cAMP production through Gαi (33). In the cell systems examined, GPR183-mediated chemotaxis also requires β-arrestin coupling but can be dissociated from receptor internalization (8).

The composition of GPR183 downstream signaling varies with cell type and experimental conditions. In primary human monocyte-derived macrophages, GPR183 stimulation induces Ca²^+^ mobilization and directional migration (34). In hepatocyte models, Gi/o, PI3K, p38 MAPK, and AMPK participate in 7α,25-OHC-GPR183-associated metabolic regulation (35). Thus, in addition to the Gi-adenylyl cyclase-cAMP axis, downstream responses involving Ca²^+^ mobilization, PI3K, and p38 MAPK show substantial cell-type and context dependence (34, 35).

Receptor activation is also accompanied by β-arrestin recruitment and dynamic signal regulation. In the cell systems examined, GPR183-mediated chemotaxis depends on β-arrestin-associated receptor desensitization, whereas constitutive and ligand-induced internalization involve different endocytic components. Caveolin and dynamin contribute to both constitutive and ligand-induced internalization, while clathrin is involved primarily in β-arrestin-independent constitutive internalization. β-Arrestin enhances ligand-induced receptor internalization, but internalization is not required for GPR183-mediated chemotaxis, indicating that β-arrestin-dependent desensitization and receptor internalization can be partially uncoupled (8). β-Arrestin recruitment, receptor desensitization, and internalization are therefore interrelated but partly separable processes; quantitative comparison of these processes may clarify gradient adaptation, cell-surface receptor dynamics, and signal duration.

GPR183 signaling acts in concert with other chemotactic axes to determine immune-cell position. GPR183, CXCR5, and CCR7 sense spatial signals formed by 7α,25-OHC, CXCL13, and CCL19/CCL21, respectively. Compound genetic-deficiency models show that GPR183 can direct chemotaxis independently of CXCR5 and CCR7 while cooperating with their positioning signals to regulate B-cell distribution across the outer follicle, interfollicular regions, and germinal-center-associated compartments (36). These ligands arise from distinct spatial sources in lymphoid tissues: 7α,25-OHC activity is enriched mainly in the outer follicle and interfollicular regions, CXCL13 maintains intrafollicular positioning, and CCL19 and CCL21 guide cells toward the T-cell zone (4, 5, 36). Integration of these parallel spatial cues enables immune-cell polarization, directional migration, and intratissue positioning (**Figure 2**). 

**Figure 2:**
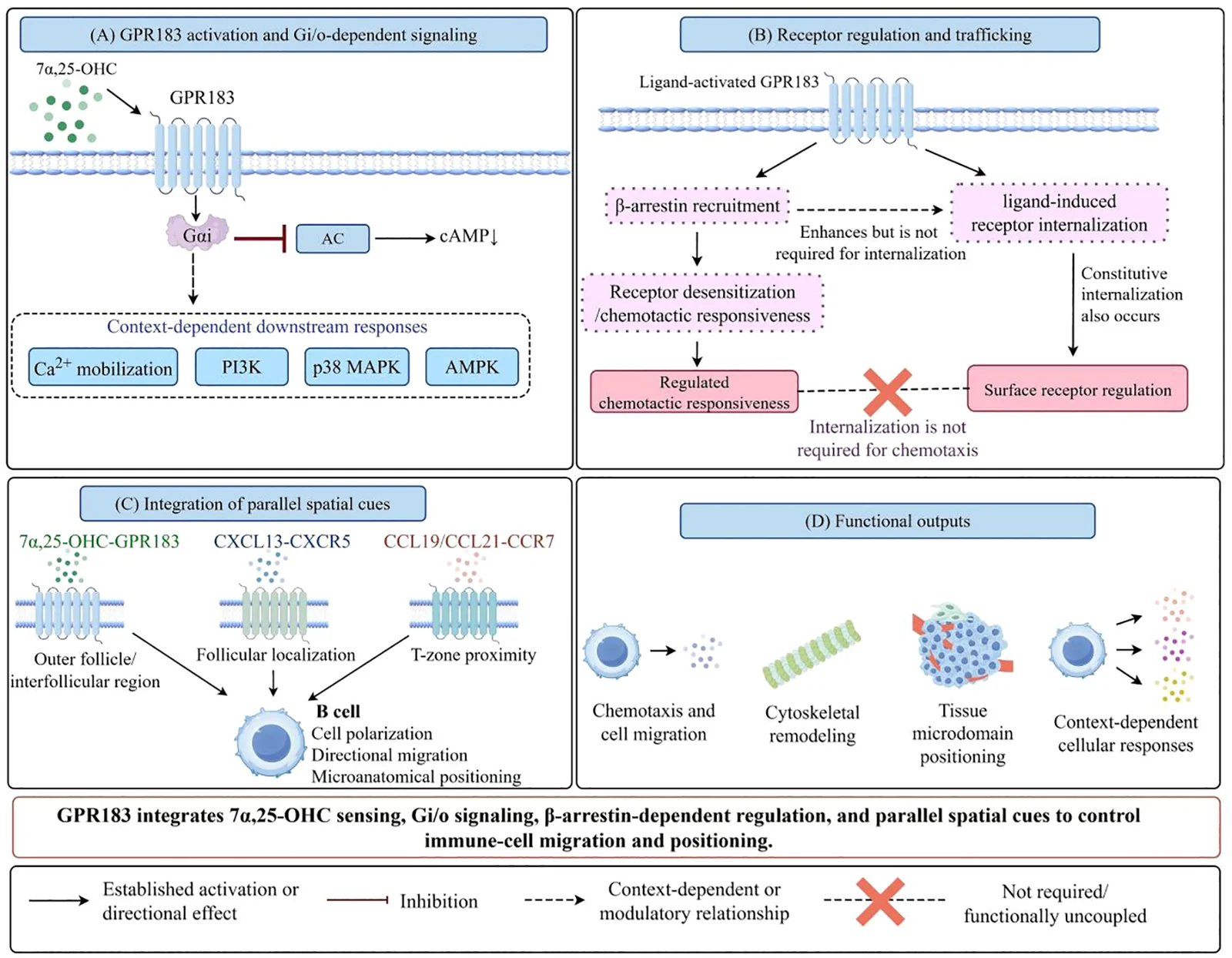
Ligand sensing, receptor regulation, and spatial positioning of immune cells by GPR183 (created with Figdraw). **(A)** 7α,25-OHC activates GPR183 and promotes Gi/o coupling; Gαi lowers intracellular cAMP by inhibiting adenylyl cyclase. Ca²^+^ mobilization and activation of PI3K, p38 MAPK, and AMPK vary with cell type and experimental conditions and therefore represent context-dependent downstream responses. **(B)** Ligand-activated GPR183 recruits β-arrestin, which contributes to receptor desensitization and regulation of chemotaxis. β-Arrestin enhances ligand-induced receptor internalization but is not required for internalization; GPR183 also undergoes constitutive internalization, thereby regulating cell-surface receptor abundance. Chemotaxis does not require receptor internalization, indicating that internalization and cell migration do not form a single obligatory pathway. **(C)** In B cells, the 7α,25-OHC-GPR183, CXCL13-CXCR5, and CCL19/CCL21-CCR7 axes provide spatial information pointing toward the outer follicle/interfollicular regions, the follicular interior, and the area adjacent to the T-cell zone, respectively. B cells integrate these parallel positioning cues to achieve polarization, directional migration, and microanatomical localization. **(D)** Integration of ligand sensing, receptor regulation, and multiple spatial cues jointly controls immune-cell chemotaxis and migration, cytoskeletal remodeling, positioning within tissue microdomains, and context-dependent cellular responses. Solid black arrows indicate established activation or directional effects, red blunt-ended lines indicate inhibition, dashed black arrows indicate context-dependent or modulatory relationships, and crossed dashed lines indicate that two processes are not obligatorily linked or can be functionally uncoupled. 7α,25-OHC, 7α,25-dihydroxycholesterol; AC, adenylyl cyclase; AMPK, AMP-activated protein kinase; cAMP, cyclic adenosine monophosphate; CCL19/CCL21, C-C motif chemokine ligands 19/21; CCR7, C-C motif chemokine receptor 7; CXCL13, C-X-C motif chemokine ligand 13; CXCR5, C-X-C motif chemokine receptor 5; Gi/o, inhibitory G-protein family; GPR183, G protein-coupled receptor 183; PI3K, phosphoinositide 3-kinase; p38 MAPK, p38 mitogen-activated protein kinase. The figure was created with Figdraw.

### 4.3 Effects of dynamic ligand gradients on immune-cell migration

Increased GPR183 ligand activity does not necessarily enhance directional migration; the outcome also depends on whether a distinct spatial differential is preserved within the tissue. Following immunization with sheep red blood cells, DCIR2^+^ cDC2s in the murine spleen upregulate GPR183 and localize primarily at the B-T border and in the outer T-cell zone. By contrast, after poly(I:C)-induced type I interferon responses, splenic *Ch25h* expression and GPR183 ligand bioactivity increase, whereas DCIR2^+^ cDC2s become diffusely distributed throughout the T-cell zone. In this setting, loss of IFNAR1 restores surface GPR183 expression on cDC2s and their localization to the outer T-cell zone, whereas *Ch25h* deficiency abolishes the poly(I:C)-induced increase in ligand bioactivity. These findings indicate that type I interferon can regulate both receptor abundance on responding cells and, through CH25H, the tissue ligand environment (37). Measurements obtained with splenic extracts and GPR183 reporter cells reflect overall tissue ligand activity rather than a spatial concentration map of 7α,25-OHC. When considered together with reduced surface GPR183 and diffuse cDC2 localization, the evidence is most consistent with broadly increased ligand activity weakening the pre-existing spatial differential and acting with receptor downregulation to reshape cDC2 positioning.

Ligand signals in the intestinal mucosa can also change reversibly with nutrient intake. A high-cholesterol diet enhances epithelial-derived GPR183 ligand signals, redistributes IgA^+^ plasma cells toward intestinal lymphatic capillaries, and is accompanied by reduced IgA secretion. Conversely, a cholesterol-free diet, inhibition of cholesterol absorption with ezetimibe, intestinal epithelial-cell-specific *Ch25h* deletion, or plasma-cell-specific *Gpr183* deletion relocates IgA^+^ plasma cells toward the intestinal epithelium and is accompanied by increased IgA secretion (10). These reversible interventions identify dietary cholesterol as an upstream determinant of the local ligand environment and link it to plasma-cell microanatomical positioning and IgA output.

Overall, GPR183-dependent migration is not simply proportional to total tissue ligand abundance. Ligand signals with discrete spatial sources and distributional differences can guide directional cell relocation, whereas broadly elevated ligand activity may weaken the directionality of an existing gradient. At the same time, GPR183 abundance on responding cells further modulates their sensitivity to changes in ligand activity (10, 37). Ligand distribution and receptor state may therefore jointly determine whether immune cells exhibit directional migration, diffuse distribution, or reduced movement.

## 5 Tissue microenvironments shape GPR183 function

### 5.1 Metabolic microenvironment

Cholesterol and its oxidized metabolites are integral components of the tissue immunometabolic environment. Nutrient input and microbial signals at barrier sites, together with systemic metabolic status, can alter the local availability of GPR183 ligands and influence how immune cells respond to tissue-positioning cues (38).

The influence of diet on GPR183-associated immune phenotypes is also evident under conditions of increased systemic metabolic load. When fed a high-fat, high-sucrose diet, female mice with global *Gpr183* deficiency show increased macrophage infiltration in visceral adipose tissue and impaired glucose tolerance but reduced hepatic inflammation and expression of macrophage-associated genes (39). Dietary cholesterol also affects GPR183-associated immune responses in a murine model of cutaneous inflammation: increased cholesterol intake aggravates Tγδ17-cell-mediated psoriasiform inflammation, and this effect requires GPR183 expression by γδ T cells (31). These models reveal tissue-specific effects of metabolic load on adipose/hepatic immunity and cutaneous Tγδ17 responses, indicating that the same nutritional input can generate different GPR183-associated phenotypes depending on the responding cell and tissue environment.

Systemic metabolic abnormalities may alter both ligand abundance and receptor status in responding cells. In a model combining diabetes with experimental autoimmune myasthenia gravis, changes in the GPR183-7α,25-OHC axis are associated with increased egress of thymic CD62L^+^ T cells; the underlying migratory mechanism is discussed in Section 5.1 (40). This composite disease model provides mechanistic evidence that metabolic dysfunction can promote T-cell egress by remodeling the thymic GPR183-7α,25-OHC axis and offers a framework for examining spatial links between metabolism and autoimmunity.

Taken together, nutritional and metabolic states can regulate the GPR183 axis through substrate availability, ligand generation, and receptor expression, whereas the responding cell and local environment determine the direction of its functional effects.

### 5.2 Inflammatory and injury microenvironments

Inflammation can reshape the local signals perceived by cells by changing the expression of 7α,25-OHC-metabolizing enzymes and GPR183 in tissue-resident populations. Systemic lipopolysaccharide stimulation differentially regulates *Ebi2*, *Ch25h*, *Cyp7b1*, and *Hsd3b7* expression in murine brain tissue and brain microvascular-associated cells. In primary murine astrocytes *in vitro*, both the CYP7B1 inhibitor clotrimazole and the GPR183 antagonist NIBR189 reduce lipopolysaccharide-induced migration, supporting the participation of GPR183 and its ligand-metabolic pathway in astrocyte migration under inflammatory conditions (41).

This regulation is more specifically defined in autoimmune inflammatory tissue. CH25H and CYP7B1 expression is elevated in skin lesions from patients with SLE and lupus-prone mice, and single-cell transcriptomic analysis identifies CXCL1^+^ fibroblasts as an important source of both enzymes. IL-1β increases CH25H and CYP7B1 expression in fibroblasts through NF-κB-p65 and p38 MAPK signaling. Conditioned medium from IL-1β-stimulated fibroblasts enhances the chemotaxis of peripheral blood mononuclear cells (PBMCs), and this response is attenuated by the GPR183 antagonist NIBR189 (42).

In an anti-CD40-induced colitis model in *Rag1*-deficient mice, colonic *Ch25h* expression increases rapidly, whereas expression of the ligand-inactivating enzyme *Hsd3b7* declines; these changes are accompanied by increased GPR183-dependent chemotactic activity in colon homogenates. GPR183^+^ innate lymphoid cells and myeloid cells accumulate within inflammatory foci. Compared with GPR183-sufficient controls, *Gpr183*-deficient mice develop fewer inflammatory foci and less severe colitis (28).

Inflammatory and injury environments can concurrently affect GPR183 ligand generation, ligand inactivation, and receptor responsiveness. This regulation may produce different positioning patterns and functional outcomes across disease settings and stages.

## 6 Changes in GPR183-related functions across disease stages and pathological states

### 6.1 Immune initiation: early immune-cell recruitment and innate activation

During immune initiation, GPR183 primarily influences the cellular composition of, and spatial contacts within, infected or inflamed tissues and draining lymphoid organs.

*Mycobacterium tuberculosis* infection provides an example of early spatial positioning. Within one week of infection, increased pulmonary eosinophil numbers are observed in both mice and nonhuman primates. Murine experiments further show that bacillus-containing alveolar macrophages upregulate CH25H and generate an oxysterol signal sensed through GPR183, thereby directing eosinophils into infected regions and toward bacillus-containing cells. Eosinophil-intrinsic GPR183 deficiency or host CH25H deficiency attenuates this recruitment (43). A similar eosinophil migratory response occurs in allergic airways. Following segmental allergen challenge in patients with mild asthma, EBI2 ligand abundance increases in bronchoalveolar lavage fluid and is associated with greater airway leukocyte accumulation. *In vitro*, 7α,25-OHC induces migration of human peripheral-blood eosinophils, and IL-5 pretreatment further enhances this response (44).

Cell-positioning effects during the initiation of infection can be dissociated from the eventual disease outcome. In influenza and SARS-CoV-2 models, GPR183 deficiency or pharmacological antagonism reduces pulmonary monocyte and macrophage infiltration and, in one SARS-CoV-2 model, is accompanied by lower disease severity and viral burden (26). In another model, however, *Gpr183* deficiency does not alter lung viral burden on day 3 of infection. NIBR189 likewise does not change early weight loss and is associated with a modest increase in lung viral burden on day 5 (45). Together, these studies place the early function of GPR183 in cellular entry and spatial organization, whereas its net effect on the host varies with pathogen, model, and observation time point.

### 6.2 Persistent inflammation: context-dependent local immune remodeling and disease effects

Time-resolved findings in M. tuberculosis infection indicate that the antimicrobial contribution of GPR183 is stage specific. In infected mice, *Gpr183* deficiency increases the pulmonary bacterial burden at two weeks, but this difference is no longer present at week five (24). This temporal pattern is consistent with antimicrobial experiments in primary human monocytes and suggests that GPR183 contributes mainly to early antimicrobial responses before adaptive immunity is fully established.

Persistent intestinal inflammation further illustrates that local tissue changes and overall disease severity do not invariably move in parallel. In acute and chronic dextran sulfate sodium (DSS) colitis, neither *Gpr183* nor *Ch25h* deficiency substantially alters disease severity. *Gpr183*-deficient mice form markedly fewer colonic lymphoid structures at steady state, and inflammation-induced accumulation of these structures is also limited. In the *Il10*-deficient model, however, *Gpr183* deficiency ameliorates colitis only in male mice, with no comparable effect in females (30). These observations distinguish local lymphoid architecture from overall disease severity as two related but non-equivalent outcomes: GPR183 can alter local immune organization, whereas its effect on colitis severity depends on the model and biological sex.

### 6.3 Tissue injury and repair: regeneration, remodeling, and immune restoration

Studies of recovery from tissue injury place GPR183-associated effects along two axes: myelin restoration and inflammatory-fibrotic remodeling. Demyelination and kidney-injury models reveal distinct repair or remodeling functions of receptor signaling in different tissue settings.

In the cuprizone-induced demyelination model, the stable analogue CF_3_-7α,25-OHC promotes remyelination of the corpus callosum, whereas natural 7α,25-OHC does not confer the same benefit, indicating different tissue effects of the stable analogue and endogenous ligand. This process is accompanied by changes in cerebral *Ebi2* expression, lipid synthesis, and immune-cell composition (46), suggesting that the repair phenotype involves tissue responses at several levels. In an earlier organotypic cerebellar-slice model of lysophosphatidylcholine (LPC)-induced demyelination, 7α,25-OHC attenuates demyelination; this effect is absent in *Ebi2*-deficient slices and is blocked by NIBR189, while *Ebi2* deficiency is also associated with delayed myelin basic protein expression (47). The LPC model focuses on demyelination and changes in myelin proteins, whereas the cuprizone model assesses post-injury remyelination. Together, they relate GPR183/EBI2 signaling to myelin injury and recovery at different tissue stages.

In contrast, global *Gpr183* deficiency reduces renal accumulation of inflammatory macrophages and tissue fibrosis and is accompanied by improved kidney function in adenine- and folic-acid-induced injury models (48). This protective phenotype encompasses both immune-cell recruitment and tissue injury, indicating that GPR183 contributes to the broader regulation of renal inflammation and tissue remodeling.

### 6.4 Tumor-associated states: cell-of-origin- and treatment-dependent immune remodeling

The role of GPR183 in cancer depends markedly on the cell expressing the receptor and the treatment context. Current evidence can be divided into malignant-cell-intrinsic effects, immune-cell states at metastatic sites, and treatment-induced microenvironmental responses.

GPR183 overexpression in non-small cell lung cancer cells suppresses proliferation, migration, and invasion and reduces lung and brain metastatic burden in mice, suggesting that the receptor may constrain metastasis-associated phenotypes in this specific tumor-cell setting (49). This is a malignant-cell-intrinsic effect. By contrast, in ovarian metastases of hormone-receptor-positive breast cancer, GPR183 is enriched primarily in a tumor-associated macrophage-like population and is associated with patient outcome. These data define the cellular source of GPR183 expression and its clinical association; receptor-perturbation studies are needed to determine whether GPR183 functionally contributes to macrophage reprogramming or metastasis (50). Treatment exposure represents a third state. In a preclinical model of Burkitt lymphoma, a regimen combining TG-1801, ublituximab, and umbralisib upregulates GPR183 in lymphoma cells. Genetic deletion of GPR183 or treatment with NIBR189 reduces macrophage infiltration, antibody-dependent cellular phagocytosis, and tumor control in the model (51).

Malignant-cell overexpression, patient single-cell data, and treatment models therefore address different questions: cell-intrinsic effects, cellular source and clinical association, and receptor dependence under a specific therapeutic regimen, respectively. The receptor-expressing cell and treatment context are consequently two central coordinates for interpreting the direction of GPR183-associated effects in cancer.

## 7 Disease-related immune functional patterns mediated by GPR183

### 7.1 Recruitment of pathogenic immune cells

Under physiological conditions, GPR183 regulates immune-cell positioning by sensing 7α,25-OHC gradients. In specific autoimmune models, GPR183-associated migration may encompass at least two spatial processes: entry of activated immune cells into damaged tissue (52) and egress of potentially pathogenic cells from central lymphoid organs to the periphery (40). The direction of its effects cannot be inferred from increased GPR183 expression alone, but depends on the ligand source, responding cell, and temporal window in which the pathway operates.

The first process is observed in experimental autoimmune encephalomyelitis. During disease, microglia upregulate CH25H and infiltrating immune cells express CYP7B1, together increasing 7α,25-OHC within the central nervous system (CNS); IL-23 and IL-1β maintain GPR183 expression in differentiating Th17 cells. After transfer of *Gpr183*-deficient encephalitogenic CD4^+^ T cells into recipient mice, fewer cells enter the CNS early and disease onset is delayed, although the difference diminishes at later time points and is not reproduced to the same extent in an active-immunization model (53). In a human blood-brain barrier model, NIBR189 reduces the attachment of CD4^+^ T cells from patients with multiple sclerosis to tri-cellular blood-brain barrier spheroids, extending GPR183-associated T-cell responses to interactions between inflammatory cells and the CNS barrier (54). Because this *in vitro* model measures attachment at the blood-brain barrier interface, it supports a role for GPR183 in early inflammatory-cell-barrier interactions rather than recapitulating complete transmigration *in vivo*.

A second process occurs when immune cells leave central lymphoid organs. In a model combining diabetes with experimental autoimmune myasthenia gravis, thymic CH25H and CYP7B1 expression and GPR183 abundance on CD62L^+^ T cells increase, accompanied by enhanced migration toward 7α,25-OHC. Pharmacological blockade of GPR183 retains more CD62L^+^ T cells in the thymus, reduces their peripheral output, and is accompanied by an attenuated myasthenic phenotype (40). The experimental autoimmune encephalomyelitis model primarily captures the migration of pathogenic T cells toward CNS lesions, whereas the combined diabetes-myasthenia model examines T-cell egress from the thymus to the periphery. Within their respective animal models, genetic and pharmacological interventions thus identify two spatial routes through which GPR183 may facilitate pathogenic-cell movement: entry into peripheral lesions and export from central lymphoid organs.

### 7.2 Recruitment of protective immune cells

During infection or tissue injury, GPR183-mediated migration can position immune cells within tissue compartments suited to pathogen clearance, barrier maintenance, and adaptive immune responses. Protection depends on whether cells reach the appropriate functional microdomain rather than on a simple increase in cell number.

When canonical CCL21-CCR7 homing signals are attenuated in inflamed lymph nodes, high endothelial venules upregulate CH25H. This enzyme converts cholesterol to 25-OHC, providing substrate for CYP7B1-mediated synthesis of the GPR183 ligand 7α,25-OHC. Naive lymphocytes sense this local signal through GPR183 and thereby retain the capacity to enter draining lymph nodes. Accordingly, GPR183 deficiency in naive lymphocytes or conditional *Ch25h* deletion in endothelial cells reduces the efficiency of lymphocyte entry into inflamed nodes; endothelial *Ch25h* deletion is also accompanied by reduced expansion and activation of antigen-specific CD4^+^ T cells (55). Locally generated 7α,25-OHC thus functions as an alternative chemotactic cue linking a vascular entry site to the ensuing adaptive immune response.

In the intestinal mucosa, GPR183 has a greater influence on intratissue cellular organization. *Gpr183* deficiency disrupts ILC3 distribution in mesenteric lymph nodes, reduces their intestinal accumulation, and lowers the number of IL-22-producing ILC3s, thereby increasing susceptibility to enteric bacterial infection (29). In mixed bone-marrow chimeras, wild-type and *Gpr183*-deficient ILC3s share the same host environment, yet the positioning defect remains largely confined to the deficient cells, indicating dependence on cell-intrinsic GPR183 expression (29). The protective effect is therefore explained more readily by positioning ILC3s where they can encounter microbial signals and exert antimicrobial functions than by a simple increase in infiltration.

Peritoneal B1 cells provide a cell-specific counterexample. Although these cells express GPR183 and respond to 7α,25-OHC *in vitro*, *Gpr183* deficiency does not substantially affect their accumulation in the omentum after lipopolysaccharide stimulation or diminish antibody responses to *Streptococcus pneumoniae* or NP-Ficoll; the more consistent change is a reduction in B2-cell abundance in the peritoneal cavity and omentum (56). Thus, GPR183 can guide immune cells to response-supporting positions in inflamed lymph nodes and the intestinal mucosa, whereas peritoneal B1-cell accumulation and antibody responses show limited dependence on this pathway.

### 7.3 Cellular mechanisms of tissue repair and remodeling

GPR183 influences tissue repair and remodeling through three types of cellular process: repopulation of immune niches, changes in tissue-resident cell states, and positioning of remodeling-associated cells.

During immune-niche repopulation, conditional depletion of alveolar or interstitial macrophages makes lung fibroblasts an important local source of 7α,25-OHC, which directs GPR183-expressing monocytes toward vacant macrophage niches. Competitive bone-marrow chimera experiments further show that *Gpr183*-sufficient cells become progressively enriched beginning at the CD64^+^ monocyte-to-macrophage transition and subsequently constitute a greater proportion of newly formed alveolar and interstitial macrophages. This competitive advantage across both transitional and mature populations links early GPR183-dependent niche entry to subsequent macrophage repopulation (57).

GPR183 can also influence tissue remodeling by altering the state of tissue-resident cells. In deoxycorticosterone acetate (DOCA)/salt- and angiotensin II-induced models of hypertension, vascular endothelial GPR183 expression increases together with endothelial senescence and dysfunction. Endothelial-cell-specific *Gpr183* deletion attenuates these changes and ameliorates hypertension-associated cardiac and renal injury; NIBR189 produces similar effects. Mechanistically, GPR183 promotes endothelial senescence by downregulating the circadian regulator PER1 and altering cAMP/PKA/CREB signaling (58). This model defines vascular injury and remodeling during chronic hypertension rather than tissue regeneration after acute injury.

Evidence that GPR183 positions remodeling-associated cells also comes from bone. Osteoblast-derived 7α,25-OHC directs GPR183-high osteoclast precursors toward bone surfaces. Intravital imaging and genetic experiments show that *Gpr183* deficiency reduces precursor motility, clustering, and fusion, limits the formation of large osteoclasts, and increases bone mass in mice; impaired GPR183 signaling also attenuates bone loss caused by aging or estrogen deficiency (59). This mechanism is not a unidirectional tissue-repair effect. Rather, GPR183 regulates bone resorption by altering the spatial distribution of osteoclast precursors and thereby contributes to the balance of bone remodeling.

### 7.4 Tumor-immune remodeling

Current studies of tumor-immune remodeling form two complementary lines of evidence: analyses of endogenous myeloid cells reveal tumor-associated state changes, whereas engineering of effector lymphocytes directly tests the tumor-navigation capacity of GPR183 in preclinical models.

Microglia in glioblastoma provide evidence for the first setting. In the murine GL261 model, microglia that take up tumor-derived extracellular vesicles downregulate *Gpr183* and several other environmental-sensing receptors while upregulating PD-L1, PD-L2, and matrix metalloproteinases. Injection of glioma-derived extracellular vesicles into the brains of healthy mice induces a subset of similar transcriptional changes (60).

Effector-lymphocyte engineering, by contrast, directly tests GPR183-mediated navigation. Introduction of GPR183 into HER2-targeted chimeric antigen receptor (CAR)-T cells enhances chemotaxis toward tumor-derived 7α,25-OHC, intratumoral infiltration, and tumor control; disruption of *Ch25h* or *Cyp7b1* in tumor cells attenuates the migratory advantage (61). An independent *in vitro* and *in vivo* CRISPR-activation screen in breast and ovarian cancer models also identified GPR183 and validated tumor tropism in NK cells, primary T cells, and their corresponding CAR-engineered derivatives. Engineered cells show increased infiltration and tumor control in xenograft models, and murine T cells overexpressing GPR183 display enhanced tumor clearance in immunocompetent models (62).

### 7.5 Biomarkers

GPR183 expression in peripheral blood and diseased tissues changes with immune-cell composition, inflammatory state, and treatment response. Relevant studies have focused primarily on associations with disease activity, therapeutic response, and prognosis. Because distinct sampling compartments capture different biological information, findings should be interpreted in relation to their source.

Studies of disease status encompass whole blood, peripheral blood mononuclear cells, and defined immune-cell subsets. Whole-blood GPR183 mRNA levels are lower in patients with tuberculosis and type 2 diabetes than in patients without diabetes; in correlation analyses across both groups, lower GPR183 expression is associated with higher chest radiographic severity scores (24). PBMCs and CD14^+^ monocytes from patients with SLE likewise show reduced GPR183 expression, which is linked to type I interferon-associated immune dysregulation (63). In lymphocyte-subset analyses, GPR183 expression in peripheral T and B cells is negatively correlated with SLE Disease Activity Index scores and positively correlated with serum C3, C4, and C1q concentrations. Receiver operating characteristic analysis also indicates that GPR183 expression in CD27^-^IgD^+^ B cells has some capacity to discriminate disease activity (64). Single-cell transcriptomic and public-cohort analyses in sepsis include GPR183 within a nine-gene candidate signature, and altered expression is also associated with abnormalities in peripheral immune-cell composition (65).

With respect to treatment response, GPR183 transcript levels in the intestinal mucosa are higher in patients with inflammatory bowel disease than in healthy controls and are further increased in nonresponders to anti-tumor necrosis factor therapy. When considered together with animal models in which disease severity does not change consistently after *Gpr183* deletion, current evidence more clearly supports GPR183 as a correlate of local inflammatory state and anti-tumor necrosis factor response than as a direct pathogenic driver (66). Research in non-small cell lung cancer provides an observation with a more clearly defined cellular source. Among patients receiving neoadjuvant immunochemotherapy, GPR183 is enriched primarily in tumor-infiltrating memory B cells and is associated with a better treatment response; this finding was subsequently validated in additional patient cohorts by multiplex immunofluorescence and flow cytometry (67). These two disease settings link GPR183 expression to treatment response in the contexts of mucosal inflammation and tumor-infiltrating B cells, respectively.

Prognostic research has used both composite models and single-gene measures. A 16-gene immune-risk score for diffuse large B-cell lymphoma includes GPR183; because independent cohorts validate the survival-stratification performance of the complete model, this evidence applies to the composite immune-risk signature rather than to GPR183 alone (68). By contrast, among 283 patients with cytogenetically normal acute myeloid leukemia, higher GPR183 transcript levels are associated with shorter overall and event-free survival, and analyses of multiple variables and an independent public cohort of 104 patients yield associations in the same direction (69). The concordant associations across discovery and validation cohorts support further evaluation of GPR183 as a component of prognostic stratification in cytogenetically normal acute myeloid leukemia.

Clinical associations of GPR183 therefore extend across disease activity, treatment response, and prognosis. Their interpretation should integrate expression level with cellular source and tissue compartment. In tissue and single-cell data, distinguishing the proportion of GPR183-positive cells from per-cell expression intensity can help determine whether an observed change primarily reflects cell composition or cell state. **Figure 3** summarizes GPR183-mediated disease-related immune functional patterns, and **Table 1** maps the strength of evidence to specific biological claims and disease settings.

**Figure 3:**
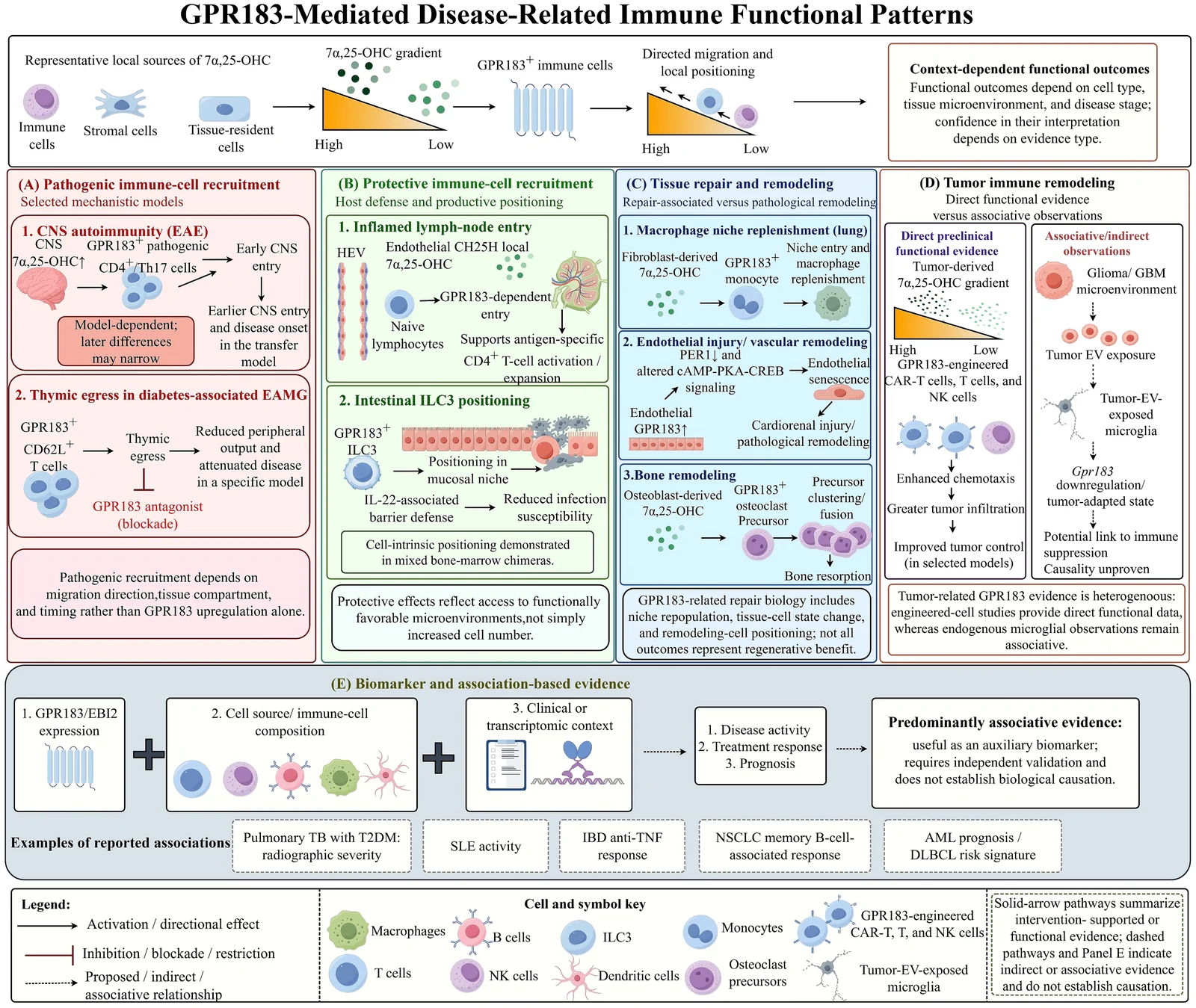
GPR183-mediated disease-related immune functional patterns (created with Figdraw). Local 7α,25-OHC gradients guide the positioning of GPR183-expressing immune cells, with functional outcomes shaped by cell type, tissue context, and disease stage; evidence type determines confidence in their interpretation. **(A)** Selected models of pathogenic immune-cell recruitment, including CNS entry in EAE and thymic egress in diabetes-associated EAMG. **(B)** Protective positioning during inflamed lymph-node entry and intestinal ILC3-mediated barrier defense. **(C)** GPR183-related processes in pulmonary macrophage-niche replenishment, vascular injury, and bone remodeling. **(D)** Direct preclinical evidence from GPR183-engineered immune cells and associative observations in tumor-EV-exposed microglia. **(E)** Association-based evidence concerning disease activity, treatment response, and prognosis. Solid black arrows indicate established activation or directional effects, red blunt-ended lines indicate inhibition or blockade, and dotted arrows indicate proposed, indirect, or associative relationships. AML, acute myeloid leukemia; CAR-T, chimeric antigen receptor T cell; DLBCL, diffuse large B-cell lymphoma; EAE, experimental autoimmune encephalomyelitis; EAMG, experimental autoimmune myasthenia gravis; EV, extracellular vesicle; GBM, glioblastoma; HEV, high endothelial venule; IBD, inflammatory bowel disease; ILC3, group 3 innate lymphoid cell; NSCLC, non-small cell lung cancer; SLE, systemic lupus erythematosus; T2DM, type 2 diabetes mellitus; TB, tuberculosis.

**Table 1:** Evidence strength for key biological and disease-related roles of GPR183.

Biological claim/disease context	Primary evidence source	Evidence strength	Key basis	Evidence boundary/priority for further validation	References
B-cell and Tfh-cell positioning during germinal-center responses	Genetic perturbation and tissue-positioning studies	High	Dynamic GPR183 expression regulates early extrafollicular positioning and the subsequent entry of activated B cells and Tfh cells into germinal-center regions.	Core functional evidence comes from murine genetic and positioning studies; human studies primarily define expression and localization.	(5–7, 9, 15–17)
GPR183-dependent cDC2 positioning	Genetic and tissue-positioning studies	High	GPR183 regulates cDC2 positioning within splenic microdomains and contributes to antigen surveillance and initiation of immune responses.	Evidence is established for murine splenic cDC2s; extension to other tissues and DC states requires validation.	(20, 37)
Immune-cell recruitment during inflammation and infection	Genetic deletion and pharmacological intervention	Moderate to high	GPR183 regulates the recruitment or positioning of myeloid cells, eosinophils, and pathogenic T cells in specific models of infection and inflammation.	Conclusions apply to defined cells, tissues, and disease stages; these variables jointly determine the direction of the effect.	(25, 26, 43–45, 52–54)
Tumor homing of GPR183-engineered T/NK cells	Cell engineering and preclinical tumor models	Moderate	Increasing GPR183 expression enhances chemotaxis, tumor infiltration, and tumor control in specific CAR-T, T-cell, and NK-cell models.	Validation is currently limited to preclinical studies; longitudinal safety and clinical efficacy are priorities for further evaluation.	(61, 62)
Clinical associations with disease activity and treatment response	Patient cohorts and clinical association studies	Low to moderate	GPR183 expression is associated with disease activity or treatment response in tuberculosis, SLE, and non-small cell lung cancer.	Current evidence supports clinical association; utility depends on prospective validation and standardized thresholds.	(24, 63, 64, 67)
Bioinformatic and prognostic associations	Transcriptomic, public-dataset, and prognostic-model analyses	Low	GPR183 is included among candidate features associated with outcomes in sepsis, diffuse large B-cell lymphoma, and other diseases.	The evidence applies to candidate features or composite models; the independent contribution of GPR183 requires direct analysis.	(65, 68, 69)

Evidence-strength criteria: Evidence strength was qualitatively assessed according to the directness of the evidence, degree of mechanistic support, and limitations related to the experimental or clinical context. High indicates direct functional evidence with strong mechanistic support, typically supported by genetic perturbation or tissue-positioning studies; Moderate to high indicates direct intervention-supported functional evidence with limitations related to context dependence or model scope; Moderate indicates functional evidence supported mainly by preclinical studies with limited translational validation; Low to moderate indicates predominantly associative evidence from clinical or observational studies with limited causal inference; and Low indicates mainly associative transcriptomic, bioinformatic, or prognostic evidence without direct functional validation.

## 8 Targeting GPR183: therapeutic strategies and translational challenges

### 8.1 Pharmacological and molecular strategies

Small-molecule development for GPR183 has proceeded along three related paths: receptor agonism, pharmacological inhibition, and signal-selective modulation. The oral antagonist IPG11406 has entered early clinical evaluation (70–72), whereas agonists, other antagonists, and biased ligands remain under preclinical optimization focused on ligand recognition, metabolic stability, *in vivo* pharmacology, and signal selectivity. Because GPR183 exhibits constitutive activity, some compounds described as antagonists also reduce basal signaling in specific assay systems and are more accurately classified as inverse agonists. Their pharmacological properties therefore cannot be defined solely by whether they block 7α,25-OHC-induced cell migration (73, 74).

For agonists, current structural optimization has focused primarily on improving the metabolic stability of 7α,25-OHC. Fluorination increases ligand stability, whereas nonsteroidal agonists derived from the NIBR189 scaffold demonstrate that the chemical space of GPR183 ligands is not restricted to steroid-like structures (75, 76). At present, the main contribution of agonist studies is therefore to expand the chemical space of GPR183 ligands and improve oxysterol stability.

The development of inhibitory ligands has progressed from research tools toward lead optimization. GSK682753A and NIBR189 were initially used to dissect constitutive signaling and ligand-induced receptor activation, respectively (73, 74). Building on this work, compound 32 combines favorable aqueous solubility, selectivity, and pharmacokinetic properties and shows *in vivo* activity at relatively low doses in a murine model of collagen-induced arthritis (77). The subsequently reported benzo[d]thiazole compound 33 has subnanomolar potency in a recombinant-cell cAMP assay and attenuates DSS-induced experimental colitis after oral administration (78). These advances provide a foundation for further chemical optimization of GPR183 inhibitors. Short-term pharmacodynamic studies in rodents support progression from tool compounds toward optimized leads and define clear next steps, including long-term immune-safety assessment, cross-species pharmacology, and evaluation in combination regimens.

Evaluation of GPR183 ligands must also account for the signaling readout and cellular background. Receptor-mediated chemotaxis requires β-arrestin coupling but not receptor internalization, indicating that Gi/o activity, β-arrestin recruitment, and migration cannot be treated as interchangeable end points (8). Pyrazolopyridazinone compound 78, reported in 2026, acts as a GPR183 inverse agonist in recombinant systems, reducing basal Gi signaling and inhibiting both 7α,25-OHC-induced Gi activation and β-arrestin2 recruitment (79). This activity was also demonstrated in PBMCs from healthy donors: compound 78 concentration-dependently inhibits 7α,25-OHC-induced migration of CD4^+^ T cells and B cells with potency similar to that of NIBR189, without substantially affecting CCL5-mediated migration or cell viability (79). Differences in the potency of compound 78 between recombinant receptor-signaling and primary-cell migration assays, its binding activity at GABA_A_ receptors, and its Y260F^6.51^-dependent Gi-biased activity together identify priorities for further selectivity optimization and structure-signaling studies.

### 8.2 Design and long-term evaluation of GPR183-engineered immune cells

Preclinical GPR183-engineering studies have demonstrated short-term enhancement of effector-cell chemotaxis and tumor homing, whereas longitudinal single-cell data collected after patient infusion associate receptor expression with the state of long-persisting CAR-T cells. Together, these two evidence streams define a dual evaluation framework encompassing short-term tumor navigation and long-term cell state.

Longitudinal data after patient infusion provide a distinct layer of evidence. In a 2023 longitudinal single-cell cohort of children with leukemia, persistent CD19 CAR-T cells gradually formed a population dominated by CD4^-^CD8^-^ cells and shared a transcriptional signature that included GPR183, TIGIT, FXYD2, and HMOX1 (80). A 2026 long-term follow-up of patients with B-cell lymphoma further showed that some CD19 CAR-T cells persisted for nearly 10 years after infusion. In-depth analysis of one patient at 9.3 years similarly identified a late transcriptional program dominated by CD4^-^CD8^-^ effector-memory-like cells and accompanied by increased GPR183 expression (81). The recurrence of this transcriptional feature across cohorts places GPR183 among candidate molecular features associated with long-term CAR-T-cell persistence. Receptor overexpression, deletion, and temporally controlled expression experiments are needed to distinguish a state marker from a functional regulator.

Evaluation of GPR183-engineered effector cells should address both tumor-navigation capacity and the long-term consequences of sustained expression. Migration in dynamic gradients and intratumoral infiltration capture navigation, whereas surface receptor density, cell state, long-term persistence, and distribution in nontarget tissues reveal how continuous expression affects cellular behavior. Stable, transient, and inducible expression create distinct windows of receptor exposure and offer different design options for balancing short-range homing with long-term cellular function.

### 8.3 Key issues and future directions for clinical translation

The central challenge in translating GPR183-directed interventions is not a binary choice between agonism and antagonism, but the development of strategies matched to cell type, tissue compartment, and disease stage. GPR183 has a strongly time-dependent role in humoral immunity: receptor deficiency disrupts the normal positioning of activated B cells and early antibody responses, whereas sustained expression impairs germinal-center-associated humoral responses (5, 6, 82). Interventions should therefore be calibrated in both magnitude and duration to avoid disrupting activated B-cell positioning and physiological antibody responses. In settings of abnormally amplified germinal-center activity, short-acting, reversible antagonism could in principle limit pathological humoral immunity, but this approach requires direct evaluation. Immunoglobulin concentrations, antibody-affinity maturation, and vaccine responses provide relevant measures of effects on normal humoral function.

GPR183 also contributes to immune positioning in mucosal and antimicrobial defense. *Gpr183* deficiency disrupts ILC3 distribution within gut-associated lymphoid tissues and increases susceptibility to intestinal infection (29), and the pathway is linked to myeloid-cell recruitment and antimicrobial responses during tuberculosis (24, 25). These functions delineate potential immune-safety boundaries for prolonged antagonism. Infection incidence, mucosal barrier function, immunoglobulin concentrations, and vaccine responses together provide a framework for evaluating the immune safety of long-term treatment.

The spatial distribution of 7α,25-OHC is maintained by local production and rapid inactivation, and this localized metabolic mechanism shapes the development pathway for ligand-based therapeutics (4). The natural ligand has low *in vivo* stability, whereas fluorination can improve stability while retaining activity (75). Local delivery or controlled release may prolong effective exposure while limiting stimulation of nontarget tissues; any advantage over systemic administration will need to be established through direct comparison.

The signaling outputs of GPR183 are not completely synchronized. β-Arrestin contributes to chemotaxis and desensitization but can be partially uncoupled from receptor internalization (8). Biased ligands must therefore be evaluated across G-protein activation, β-arrestin recruitment, and cell-migration end points rather than judged on a single signaling change. Direct comparison of ligands with different signaling biases is required to determine whether pathological recruitment can be controlled while protective immunity is retained.

As of 16 August 2026, all GPR183-related interventional studies registered on ClinicalTrials.gov involved the oral antagonist IPG11406. The Phase I study NCT06255834 in healthy volunteers has been completed and primarily evaluated safety, tolerability, and pharmacokinetics (70). The Phase Ib/IIa study NCT06717815 in lupus nephritis is recruiting (71). The Phase II study NCT07535489 in ulcerative colitis has been registered but is not yet recruiting (72). IPG11406 has therefore made oral antagonism the first GPR183-directed strategy to enter clinical evaluation, whereas agonists, biased ligands, and tissue-targeted formulations represent differentiated directions for subsequent development.

Future development should integrate patient stratification, tissue exposure, and immune safety. CH25H and CYP7B1 expression and local 7α,25-OHC abundance may be included as exploratory measures of pathway activity, while tissue drug concentration, receptor occupancy, and changes in cell migration may serve as candidate pharmacodynamic measures. Reversible antagonism, local delivery, and validated biased-modulation strategies may help control inflammation while limiting disruption of physiological immune function, although each approach requires direct comparative evidence. Defining therapeutic windows and immune-recovery profiles in individual diseases will enable more accurate assessment of the long-term benefits and risks of GPR183-directed treatment. **Figure 4** summarizes the progression of GPR183-targeted agents from preclinical pharmacology to early clinical evaluation and highlights translational design considerations, including tissue exposure, pharmacological selectivity, and long-term immune safety.

**Figure 4:**
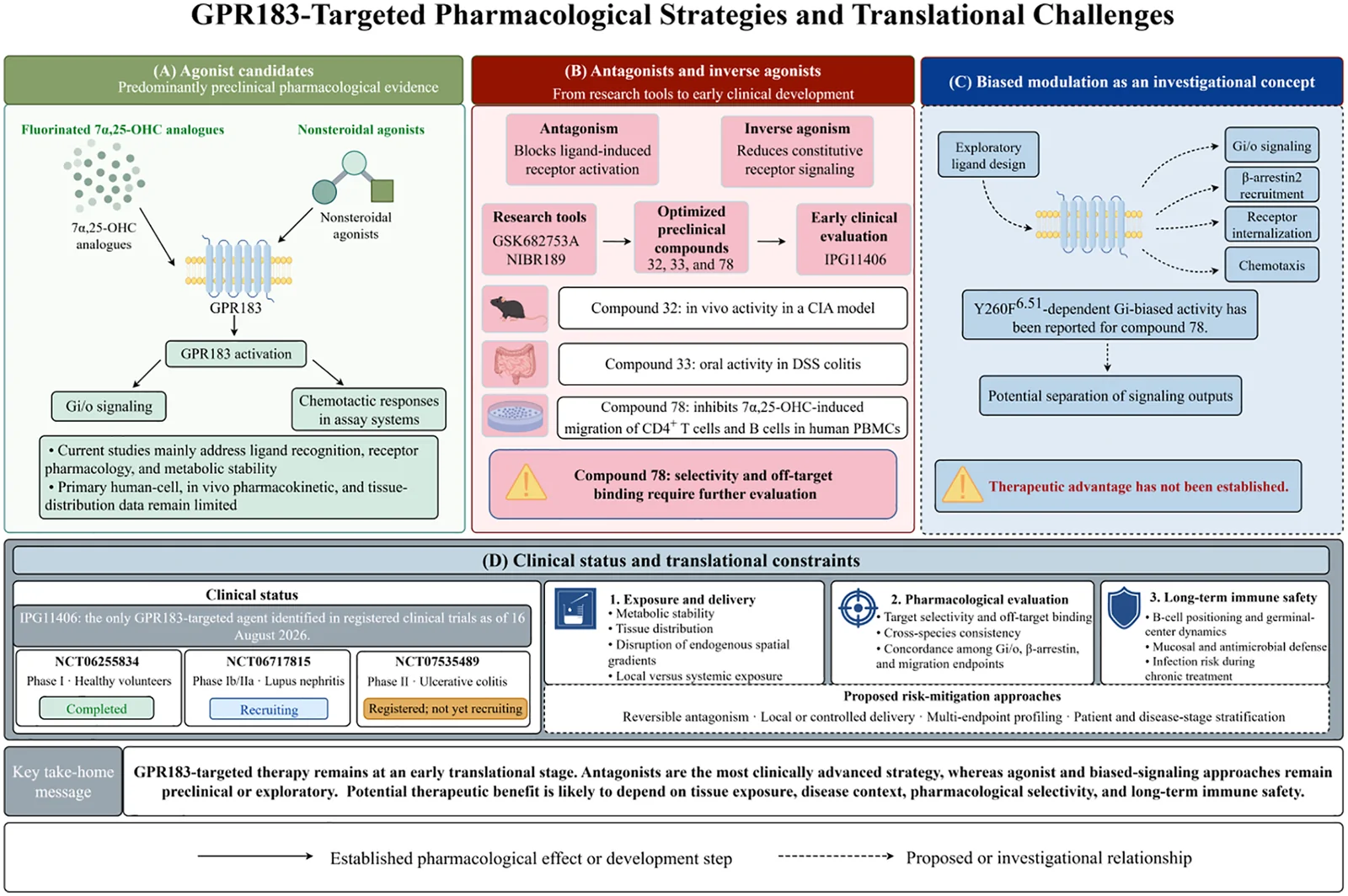
GPR183-targeted pharmacological strategies and translational challenges (created with Figdraw). **(A)** Agonist candidates. Fluorinated 7α,25-OHC analogues and nonsteroidal agonists activate GPR183 and have been evaluated principally for ligand recognition, receptor pharmacology, metabolic stability, and chemotactic activity in assay systems; human-cell, *in vivo* pharmacokinetic, and tissue-distribution data remain limited. **(B)** Antagonists and inverse agonists. Research tools such as GSK682753A and NIBR189 have supported mechanistic studies, while compounds 32, 33, and 78 represent optimized preclinical inhibitors with activity in arthritis, colitis, or primary-cell migration assays. IPG11406 is the first GPR183-directed agent to enter early clinical evaluation. The selectivity and off-target binding of compound 78 require further assessment. **(C)** Biased modulation. Exploratory ligand design may differentially affect Gi/o signaling, β-arrestin2 recruitment, receptor internalization, and chemotaxis. Y260F^6.51^-dependent Gi-biased activity has been reported for compound 78, but a therapeutic advantage of signal bias has not been established. **(D)** Clinical status and translational constraints. IPG11406 has completed evaluation in healthy volunteers, is being evaluated in lupus nephritis, and is planned for evaluation in ulcerative colitis. Key development requirements include appropriate exposure and delivery, target selectivity and cross-species pharmacology, concordant profiling across signaling and migration end points, and long-term monitoring of B-cell positioning, germinal-center dynamics, mucosal defense, and infection risk. Solid arrows indicate established pharmacological effects or development steps; dashed arrows indicate proposed or investigational relationships.

## 9 Conclusion

By sensing local 7α,25-OHC gradients, GPR183 links tissue metabolic states to the spatial positioning of immune cells. Coordinated changes in receptor expression and ligand gradients, rather than receptor abundance at a single time point, determine the direction of immune-cell migration and its subsequent functional consequences. Genetic, pharmacological, and cell-engineering studies have established a mechanistic framework in which GPR183 contributes to lymphoid-tissue positioning, inflammatory recruitment, host defense, tissue remodeling, and tumor-immune navigation. Studies of human tissues and clinical cohorts, together with early clinical trials, provide disease-relevance and translational evidence for this framework.

The translational value of GPR183 lies in the stratified control of spatial immune-cell behavior. Receptor agonism, pharmacological antagonism, and engineered expression address distinct goals: enhancing protective recruitment, limiting pathological migration, and improving effector-cell navigation, respectively. Future development should match the intervention to the responding cell, target tissue, and disease stage, while combining spatial ligand quantification, reversible or local delivery, and longitudinal immune monitoring to define therapeutic windows. GPR183 is therefore best viewed not as a uniformly pro- or anti-inflammatory target, but as an immune-navigation node that may be modulated according to tissue context and disease stage.
